# Parallel subfunctionalisation of PsbO protein isoforms in angiosperms revealed by phylogenetic analysis and mapping of sequence variability onto protein structure

**DOI:** 10.1186/s12870-015-0523-4

**Published:** 2015-06-09

**Authors:** Miloš Duchoslav, Lukáš Fischer

**Affiliations:** Department of Experimental Plant Biology, Faculty of Science, Charles University in Prague, Viničná 5,, 128 44 Praha 2 Czech Republic

**Keywords:** Gene duplication, GTPase, Homology modelling, Manganese-stabilizing protein (MSP), Oxygen evolving complex, Parallel evolution, Protein structure, PsbO

## Abstract

**Background:**

PsbO, the manganese-stabilising protein, is an indispensable extrinsic subunit of photosystem II. It plays a crucial role in the stabilisation of the water-splitting Mn_4_CaO_5_ cluster, which catalyses the oxidation of water to molecular oxygen by using light energy. PsbO was also demonstrated to have a weak GTPase activity that could be involved in regulation of D1 protein turnover. Our analysis of *psbO* sequences showed that many angiosperm species express two *psbO* paralogs, but the pairs of isoforms in one species were not orthologous to pairs of isoforms in distant species.

**Results:**

Phylogenetic analysis of 91 *psbO* sequences from 49 land plant species revealed that *psbO* duplication occurred many times independently, generally at the roots of modern angiosperm families. In spite of this, the level of isoform divergence was similar in different species. Moreover, mapping of the differences on the protein tertiary structure showed that the isoforms in individual species differ from each other on similar positions, mostly on the luminally exposed end of the β-barrel structure. Comparison of these differences with the location of differences between PsbOs from diverse angiosperm families indicated various selection pressures in PsbO evolution and potential interaction surfaces on the PsbO structure.

**Conclusions:**

The analyses suggest that similar subfunctionalisation of PsbO isoforms occurred parallelly in various lineages. We speculate that the presence of two PsbO isoforms helps the plants to finely adjust the photosynthetic apparatus in response to variable conditions. This might be mediated by diverse GTPase activity, since the isoform differences predominate near the predicted GTP-binding site.

**Electronic supplementary material:**

The online version of this article (doi:10.1186/s12870-015-0523-4) contains supplementary material, which is available to authorized users.

## Background

Photosynthetic conversion of light into chemical energy in oxygenic phototrophs is accompanied with evolution of molecular oxygen released from water molecules. This process is realized in the oxygen evolving complex of photosystem II present in thylakoid membranes. Photosystem II (PSII) is a multisubunit protein–cofactor complex that uses light energy to oxidize water and to reduce plastoquinone. PsbO, also known as the manganese-stabilising protein, is one of the extrinsic subunits of photosystem II, located on the luminal side of the thylakoid membrane. PsbO is present in all known oxygenic photosynthetic organisms [1]. Despite the ability of the cyanobacterium *Synechocystis* sp. PCC 6803 mutant to grow photoautotrophically with deleted *psbO* gene [2], PsbO seems to be crucial for PSII function. Neither the mutant of green alga *Chlamydomonas reinhardtii* lacking PsbO, nor *Arabidopsis thaliana* (*A. thaliana*) with silenced expression of both *psbO* paralogs were able to grow photoautotrophically or even assemble PSII [3, 4].

Three-dimensional structure of PsbO from cyanobacterium *Thermosynechococcus* was resolved as a part of PSII by X-ray crystallography with a resolution down to 1.9 Å [5]. The crystal structure of PSII or PsbO alone from plants or other eukaryotes is not available. Some information about the structure of the whole PSII dimer surrounded by antenna complexes (the PSII-LHCII supercomplex) from higher plants was obtained by single particle cryo-electron microscopy and cryo-electron tomography [6–8]. Unfortunately, the resolution is insufficient to provide any plant-specific knowledge about the PsbO structure. Still, relatively high pairwise identity between PsbO sequences of *Thermosynechococcus* and higher plants (around 45 %) allows construction of homologous models for plant PsbOs [9, 10].

The X-ray crystallography of cyanobacterial PSII revealed that PsbO is a β-barrel protein (structural features of PsbOs are discussed in connection with our results and PsbO functions in chapter Discussion). It is located in the vicinity of the water splitting Mn_4_CaO_5_ cluster, but it is not directly involved in binding of the cluster [9]. The main function of the PsbO is to stabilise the Mn_4_CaO_5_ cluster, in particular to modulate the calcium and chloride requirements for efficient water splitting (for review see [11]). Besides this “basic” function, PsbO seems to be involved also in other processes (for review see [12, 13]). Spinach PsbO was shown to be able to bind GTP [14] and also to hydrolyse it, although very slowly [10]. It was proposed that the GTPase activity of PsbO in plants might be involved in D1 repair cycle [10].

In plants and algae, the PsbO protein is encoded by a nuclear *psbO* gene [1]. Transport to chloroplasts and thylakoids is ensured by two consecutive N-terminal transit peptides, that are cleaved to produce the mature PsbO [15]. *A. thaliana* expresses two *psbO* genes, *psbO1* [TAIR:At5g66570] and *psbO2* [TAIR:At3g50820], encoding for PsbO1 and PsbO2 proteins [16, 17]. The two isoforms differ in only 11 amino acids [18]; nevertheless, their function seems to be slightly different. Murakami *et al.* [18] reported that *A. thaliana* PsbO2 recovered oxygen evolution of PsbO-depleted spinach PSII particles less efficiently than PsbO1. The activity with PsbO2 reached only 80 % of that with PsbO1, while the binding efficiency of the isoforms was very similar. In contrast, the oxygen evolution of PSII membranes isolated from *A. thaliana* mutants lacking PsbO1 or PsbO2 was similar when corrected for the amount of PSII [19].

The amount of PsbO1 in wild-type *A. thaliana* plants is higher than that of PsbO2 [18–20]. The expression of the isoforms stays similar during plant development and during various short time stresses [21]. Only after 40 days of cold stress, noticeable change in relative abundance of isoforms was observed in favour of PsbO2 [22].

In *A. thaliana* mutants with an impaired *psbO1* or *psbO2* gene, the compensatory upregulation of the remaining isoform was observed. The expression level of PsbO2 in *psbo1* mutant was increased several times, reaching 75 % of the total amount of PsbO in wild-type. The expression level of PsbO1 in *psbo2* mutant was 125 % of the total PsbO in wild-type. The amount of other PSII proteins was affected similarly, leading to the same stoichiometry of PsbO per PSII as in wild type [19].

The *psbo1* mutant plants have pale green leaves, reduced rosette size and slower growth rate as compared to wild-type plants [17, 19, 23]. Descriptions of the *psbo2* mutant phenotype slightly differ from each other, probably because of different growth conditions and age of used plants [23]. Lundin *et al.* [19] observed growth rate slower than in wild-type and the leaf weight was even lower than that of *psbo1*, while Allahverdiyeva *et al.* [23] reported a phenotype very similar to that of wild-type.

Under growth light (120 μmol photons m^−2^ s^−1^), the *psbo2* mutant had characteristics of electron transport chain very similar to wild-type, whereas investigation of the *psbo1* mutant showed malfunction of both the donor and acceptor sides of PSII and high sensitivity of PSII centres to photodamage [23]. Bricker and Frankel [24] reported that many of the defects of *psbo1* photosystems are reverted by higher concentration of CaCl_2_, but Allahverdiyeva *et al.* [23] did not observe similar effect. Nevertheless, the importance of the PsbO2 seems to be exhibited under high light conditions. For example, the maximum quantum efficiency (F_V_/F_M_) values of wild-type and mutant plants became similar after 3 weeks of moderate light (500 μmol photons m^−2^ s^−1^) [23]. Lundin *et al.* [19] reported that after 15 days of high light (1000 μmol photons m^−2^ s^−1^), the *psbo1* mutant did not have significantly reduced leaf weight, whereas the leaf weight of *psbo2* mutant was reduced drastically.

Lundin *et al.* [19] also showed that *psbo2* mutant has lower level of phosphorylation of D1 and D2 subunits and that the degradation of photo-damaged D1 protein is impaired in this mutant. This, together with a finding that PSII membranes with PsbO2 have higher GTPase activity than PSII membranes with PsbO1 [21], led to a conclusion, that PsbO1 has a main function in the stabilisation of Mn_4_CaO_5_ cluster and the facilitation of the water oxidation reaction, whereas PsbO2 regulates the turnover of D1 subunit [19, 21, 23].

The presence of two PsbO isoforms is not unique for *A. thaliana*. Our previous study focused on the analysis of a spontaneously tuberising potato mutant revealed that potato plants also express two PsbO isoforms, one of which is missing in the mutant [25]. A comparison of the two characterised *A. thaliana* and two potato PsbO isoforms showed that sequences of the two paralogs in each species are more related than isoforms coming from different species. It indicated independent duplication of *psbO* gene in these two species. To understand this unexpected phylogeny and evolution of PsbO isoforms, we did a detailed analysis of *psbO* sequences from a number of land plant species. Mapping the sequence differences between PsbO proteins from various species and families and between PsbO isoforms in individual species on their tertiary structure, we found that the evolution of the two isoforms was parallel in numerous angiosperm lineages. Based on the location of isoform-specific differences and literature data about *A. thaliana* and spinach PsbOs, we hypothesise that the pairs of isoforms present in many species differ in GTPase activity and that the presence of proteins diversified in this way helps to improve photosynthetic performance under varying conditions.

## Materials and methods

### Retrieval and analysis of psbO sequences

Sequences of expressed *psbO* genes were retrieved as ESTs (expressed sequence tags) and assembled ESTs (PUTs, PlantGDB-assembled unique transcripts) in public sequence databases NCBI GenBank [26] and PlantGDB (Plant Genome Database) [27], respectively. The database searches were performed using tBLASTn [28, 29] with potato PsbO protein sequence (sequence “Solanum tuberosum 2”, translation of [PlantGDB:PUT-157a-Solanum_tuberosum-55973153]) as a query. ESTs were aligned into contigs for each species using “De Novo Assemble” tool of Geneious R6 [30]. Formation of consensus sequences from multiple overlapping ESTs strongly increased reliability of analysed sequences compared to individually submitted annotated cDNAs, some of which contain evident errors. All retrieved sequences were aligned using MAFFT v7.017 [31] and incomplete and unreliable sequences were excluded from further analyses (see analysed sequences in Additional file 1). Spinach *psbO* sequence was retrieved as cDNA [GenBank:X05548.1] because of the lack of ESTs and included in alignment for comparison (Additional file 2). Indexing of isoforms in each family was random and does not reflect relation to *A. thaliana* isoforms.

Phylogenetic trees were built from *psbO* coding sequences by maximum likelihood (ML) method using CIPRES Science Gateway [32]. ML analysis was implemented in tool RAxML v7.6.6 [33] using GTRGAMMA approximation with 1000 bootstrap replicates.

The presence and position of introns was analysed by comparing *psbO* cDNAs (Additional file 1) and corresponding genomic sequences, obtained using BLASTn [28, 29] searches in Phytozome database [34] for the following representative species with easily available genomic sequence: *Arabidopsis lyrata*, *Arabidopsis thaliana*, *Brassica rapa*, and *Thellungiella halophila* from Brassicaceae family and *Oryza sativa*, *Physcomitrella patens*, *Populus trichocarpa*, *Solanum lycopersicum*, and *Vitis vinifera* from other families.

### Evaluation of PsbO sequence variability

The frequency of differences between isoforms, between species and between families were calculated for each position in the alignment independently using scripts written in R language [35] and partially using SeqinR package [36]. Plant families represented with just a single PsbO sequence were not included in the calculation. Only two most divergent isoforms were considered in case of species expressing more than two isoforms. All sequences excluded from calculation are marked with an asterisk in Additional file 2. To estimate the between-isoform and between-species variability across all angiosperms, both types of differences were first calculated for every family independently and afterwards the values were averaged, in order to avoid bias caused by different numbers of analysed species within each family.

The frequency of between-isoform differences within a family was calculated as follows; first, each position in the alignment was assigned 0 or 1 (for the same or different amino acids in the two compared isoforms, respectively) for each species and then the values were averaged within a family. To get the frequency of between-species differences, all species within a family were compared pair wise with each other, giving the values 0, 0.5 or 1 (for amino acids in both isoforms identical, amino acid in one isoform identical or no identical amino acid) for each position and each comparison. Values for each position were averaged within a family. As the dependency of this average variability value on the proportion of species that have certain amino acid different from the consensus is not linear, it was linearised using the equation

$$ {\varDelta}_{species\  linear}=\frac{\left(2n-1\right)-\sqrt{4\ \left(1-{\varDelta}_{species}\right)\ n\ \left(n-1\right)+1}}{2\ \left(n-1\right)} $$

where *n* is the number of compared species, ∆_*species*_ is the non-linear average value of between-species variability (the mean from pair wise comparisons) and ∆_*species linear*_ is the linearised value of the between-species variability.

To estimate the between-family variability, the above mentioned method for the calculation of the between-species differences was applied on sets containing sequences from just one species from each family. A mean values obtained from all such combinations of species (53,760 in total) included both between-species and between-family differences, so the values of between-species differences were subtracted from it, giving the net between-family differences.

### Homology modelling and mapping of variability on the protein structure

Homology model of potato PsbO (sequence “Solanum tuberosum 2”) was built using Swiss-Model server [37, 38] based on PsbO from cyanobacterium *Thermosynechococcus vulcanus* [PDB:3ARC] (chain O) [5]. Extra 13 amino acids present on the N-terminus of potato PsbO were pasted to the model manually using Swiss-PdbViewer v4.1.0 [39] without attempt to show any folding.

Homology model of potato PsbO was coloured according to the frequency of the respective type of variability using Swiss-PdbViewer v4.1.0 [39] and scripts written in R language [35]. The images were rendered using POV-Ray v3.6 [40].

### Determination of spatial centres of differences

Spatial centres of the differences were calculated using coordinates of α-carbon atoms of amino acids in the PsbO homology model using scripts written in R language [35]. The arithmetic mean of the coordinates was weighed by frequency of the respective difference on each position. The 13 N-terminal amino acids with unknown folding were excluded from the calculation. Overall spatial centres of the differences between isoforms and the differences between species in angiosperms were calculated as an arithmetic mean of spatial centres calculated for all families. The statistical significance of the divergence in the location of the spatial centres of the between-isoform and the between-species differences was assessed using a randomisation test. Variable positions in the alignment were randomly shuffled and the spatial centres for the between-isoform and the between-species variability were calculated. Difference between means of the two types of spatial centres projected on the axis of highest variability was compared with the value obtained for real alignment. The *p*-value was calculated from 50,000 randomisations.

## Results

### The majority of angiosperm species express two *psbO* genes

Searching public databases for expressed sequences of *psbO* genes from land plants (Embryophyta) we obtained 91 sequences from 49 species and 36 genera. Analysis of these sequences showed that the majority of the analysed angiosperm species express more than one, in most cases two *psbO* isoforms (Additional file 3). In contrast, all analysed representatives of gymnosperms (from both Cycadophyta and Coniferophyta groups) seem to express only one *psbO* isoform.

In monocots, *psbO* sequences were available from only two families: Zingiberaceae species have two *psbO* isoforms, whereas most Poaceae species with available ESTs express only one *psbO* gene. A single *psbO* gene was found also in the genomic sequence of *Oryza sativa. Zea mays*, a recent tetraploid, expresses two isoforms with little divergence (Additional file 3).

Among dicots, Malvaceae, Myrtaceae, Phrymaceae and Rutaceae seem to express only one *psbO* gene. Asteraceae, Euphorbiaceae, Fabaceae, Salicaceae, Solanaceae and Vitaceae seem to express two *psbO* genes (or four in the case of recent tetraploids such as *Glycine max* or *Nicotiana tabacum*). Brassicaceae have various numbers of *psbO* isoforms; however, most of them can be sorted into two groups. While *Arabidospsis thaliana* expresses just two isoforms (*psbO1*, *psbO2*), each from one group, genus *Brassica* expresses three to five genes - one gene corresponds to *psbO2* of *A. thaliana*, while the gene orthologous to *psbO1* of *A. thaliana* is present in several very similar sub-isoforms (4 in *B. napus*, 3 in *B. rapa* and 2 in *B. oleracea*; Additional files 3 and 4). *Thellungiella halophila* expresses three *psbO* genes, two of which correspond to *psbO1* and *psbO2* of *A. thaliana*, the third one is most similar to pseudogenes that can be found in genomic sequences of *A. thaliana* [TAIR:At4g37230], *Arabidopsis lyrata* [GenBank:XM_002866937] and *Brassica rapa* [Phytozome:Bra017790] (data not shown).

### Pairs of PsbO isoforms evolved in every angiosperm family independently

**Fig. 1:**
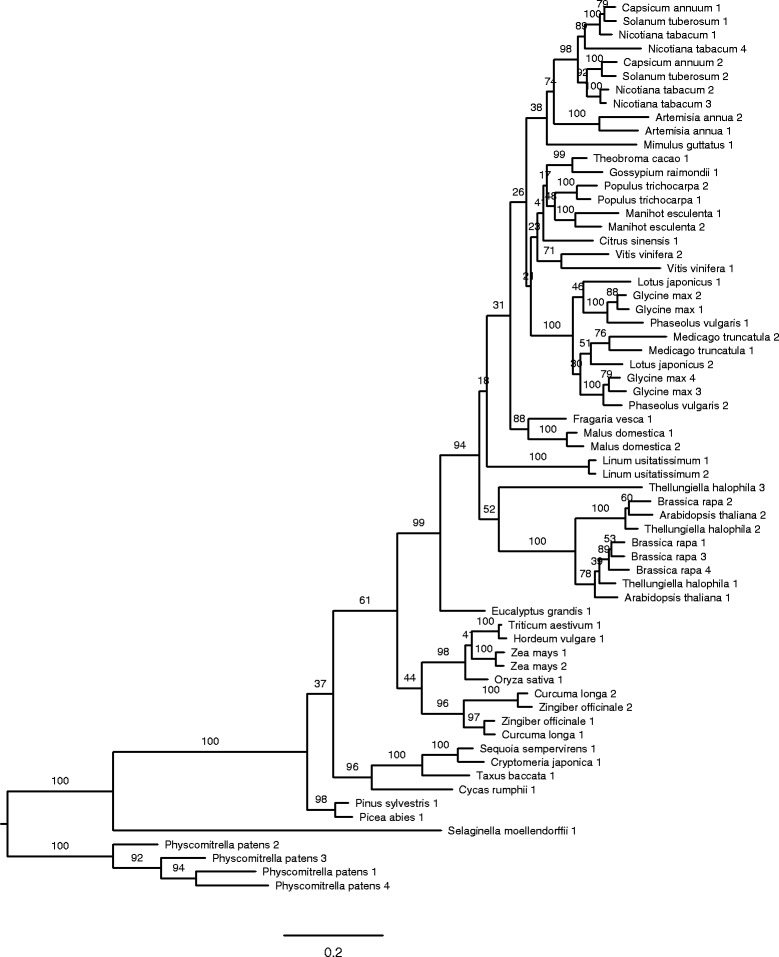
A phylogenetic tree from coding sequences of *psbO* genes from 36 genera of land plants. Each genus is represented by sequences from only one species for the sake of simplicity. Sequences from different species belonging to the same genus are very similar and their inclusion does not change the phylogenetic tree topology (see the full phylogenetic tree in Additional file 4). The tree was constructed by the maximum likelihood method, numbers at branches denote bootstrap percentages

The majority of analysed angiosperm species have just two PsbO isoforms (Additional file 3). Such situation could likely results from a gene duplication event in a common ancestor followed by functional divergence of the paralogs. The paralogous genes encoding the functionally divergent isoforms can be inherited by descendants or potentially lost. However, the phylogenetic tree derived from coding sequences of *psbOs* indicates a different evolutionary scenario (Fig. 1).

The basic topology of the phylogenetic tree does not contain dichotomous branching to two groups of functionally diverged orthologs at the tree base, but it reflects basic phylogeny of land plant families. The branching to two isoforms is also absent at the base of angiosperms. Instead, the branching events are clearly present at the bases of several families (for example Solanaceae, Fabaceae, Brassicaceae, Zingiberaceae; Fig. 1). This unexpected topology indicates that duplications of *psbO* gene occurred independently in each plant family that contains species with multiple PsbO isoforms. Moreover, these families do not form any cluster in the phylogenetic tree of *psbO* or in the consensual phylogeny of angiosperms.

To further confirm the independent duplication of *psbO* genes in ancestor of each angiosperm family, the presence and position of introns was analysed in available genomic sequences of *psbO* genes. According to this analysis, all land plants have an intron at a conserved site, 12 nucleotides upstream the boundary between sequences encoding the transit peptide and the mature protein. In addition, all *psbO* genes from Brassicaceae family contain an additional intron, 282 nucleotides downstream the boundary between the transit peptide and the mature protein. The intron is present at a conserved site in all *psbO* genes in this family, including the most divergent isoform of *Thellungiella halophila*. This indicates that all these *psbO* genes evolved from one common Brassicaceae-specific ancestor gene containing the additional intron, absent in *psbOs* in other families.

### Extent of divergence of PsbO isoforms is similar in all species

The extent of differences between protein sequences of PsbO isoforms in every species is in the same range, even though the duplication seems to have occurred in each family independently. The numbers of different amino acid residues range from six in a recent tetraploid *Zea mays* to 23 in *Populus deltoides* and *Populus x canadensis* (2–9 % of total residues; Additional file 3). Interestingly, similar divergence between isoforms can be found also in the moss *Physcomitrella patens* (24 different amino acid residues between two most divergent isoforms).

The level of differences between PsbO isoforms is kept within this range even if the duplication events of *psbO* occurred at different times in evolutionary history. For instance, pairwise identity of nucleotide sequences encoding mature PsbOs of *V. vinifera* (80 %) is much lower than that of *Populus trichocarpa* (92 %). This indicates that the duplication of the *Vitis psbO* gene probably occurred earlier compared to that of the *Populus* gene. However, pairwise identity of the protein sequences of PsbO isoforms of *V. vinifera* (93 %) is similar to that of *P. trichocarpa* (92 %).

### Three classes of PsbO sequence variability

Considering that many angiosperm species express two isoforms of *psbO*, we asked whether the differences between the isoforms are similar in multiple families despite the independent duplications of *psbO* genes. Detailed analysis of the sequence alignment failed to identify any compact region in the primary sequence that would be specific for one or the other isoform across the analysed plant families. Also, single positions with similar differences between isoforms in the majority of species were rare (see the alignment in Additional file 2).

**Fig. 2:**
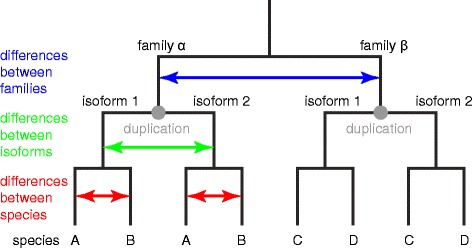
A scheme of the *psbO* phyllogeny showing three classes of PsbO sequence variability. Differences between families are in blue, differences between isoforms in green and differences between species in red

To analyse the character of the differences in PsbO sequences in detail, we assorted the variability into three classes: i) variability between isoforms (within a species), ii) variability between species (within a family) and iii) variability between families (Fig. 2). Frequencies of these three classes of variability were calculated for each position of the primary sequence (Additional file 2; see Materials and methods section for details). In the alignment of mature PsbO sequences from angiosperms (Additional file 2), 59 % of positions are fully identical, 77 % of positions can be described as conserved (with low level of variability below 10 %). The variability in the remaining 23 % of positions could stem from either selection pressure favouring a specific substitution (positive selection), or, on the contrary, from the lack of strong selection pressure to keep the position invariable (negative selection). The lack of selection pressure should result in frequent random changes and a high level of variability in all three classes. When analysing the PsbO sequences, it was obvious that a certain class of variability predominated at many positions and that the overlap between the classes at a given position was only partial (Additional file 5).

### Amino acid residues varying between isoforms differ predominantly in the length of side chains

**Fig. 3:**
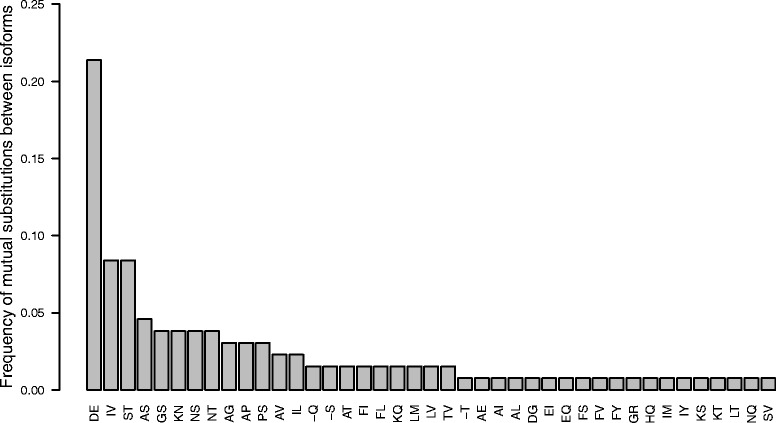
Frequency of amino acid substitutions between isoforms. The amino acid residues differing on certain position in isoform pairs of analysed species are given below the bars, the hyphen (−) represents a gap. For the analysis, one representative species with two isoforms was chosen from each family in order to avoid the bias caused by various numbers of species with available data in each family (analysed species: *Arabidopsis thaliana*, *Artemisia annua*, *Lotus japonicus*, *Malus domestica*, *Manihot esculenta*, *Populus trichocarpa*, *Solanum tuberosum*, *Vitis vinifera*, *Zea mays*, *Zingiber officinale*)

Analyses of substitutions at positions variable between isoforms showed that some substitutions were more frequent than others. The most frequent differences between isoforms resided in mutual exchanges of glutamic (E) and aspartic (D) acid residues (more than 20 % of all substitutions; Fig. 3). Distribution of these two residues within the isoform pairs was usually unequal. In PsbO pair in certain species, glutamic acid often predominated in most of variable positions in one isoform, whereas aspartic acid in the other (Additional file 6). The total number of these two residues was more or less constant. According to this distribution and the residue present on position 140 (E139 in spinach), almost each pair of isoforms could be divided into the E-type isoform (with predominating longer glutamate) and the D-type isoform (with prevailing shorter aspartate). According to this, *A. thaliana* PsbO1 clustered into D-type isoforms, whereas PsbO2 into E-type, though the divergence in D/E ratio between isoforms was not as strong as in many other species. PsbOs in the analysed species with single isoform were either closer to the E-type or to the D-type isoforms or were in the mid-way, e.g. PsbOs from Poaceae species or *Linum usitatissimum* clustered with E-type isoforms, whereas PsbOs from non-herbaceous Rutaceae or Myrthaceae species were close to D-type isoforms (Additional file 6). The D-type isoforms were also often prolonged at C-terminus with an additional amino acid residue.

Exchanges in other amino acid residues were less conserved among various families. But generally, substitutions between residues, which differed only in the length of the side chain and had similar physicochemical properties, predominated over substitutions between residues with more variable character. The three most frequent amino acid substitutions (D-E, I-V and S-T; Fig. 3) match these criteria and comprise together almost 50 % of all exchanges. Though seemingly synonymous, these substitutions are strongly conserved in orthologous isoforms within families and in some cases even shared across more families (see the alignment in Additional file 2).

### Residues varying between isoforms cluster together on the tertiary structure of PsbO

The positions with amino acids varying predominantly between isoforms did not cluster together in the primary sequence. As protein function is tightly connected with tertiary structure, we decided to analyse spatial location of amino acid substitutions between PsbO isoforms on the protein structure. Because no crystal structure of eukaryote PsbO is available, we constructed homologous model of PsbO2 from *Solanum tuberosum* using PsbO structure from *Thermosynechococcus vulcanus* [5] as a template (identity of the protein sequences is 47 %). All PsbO sequences of angiosperms are well comparable on a single model of structure thanks to a very high conservation of both the amino acid sequence and the length of the chain. In the alignment of 78 protein sequences of PsbOs from angiosperms, 59 % of positions are fully identical and the length of the chain of mature proteins varies mostly between 247 and 248 amino acid residues (Additional file 2).

**Fig. 4:**
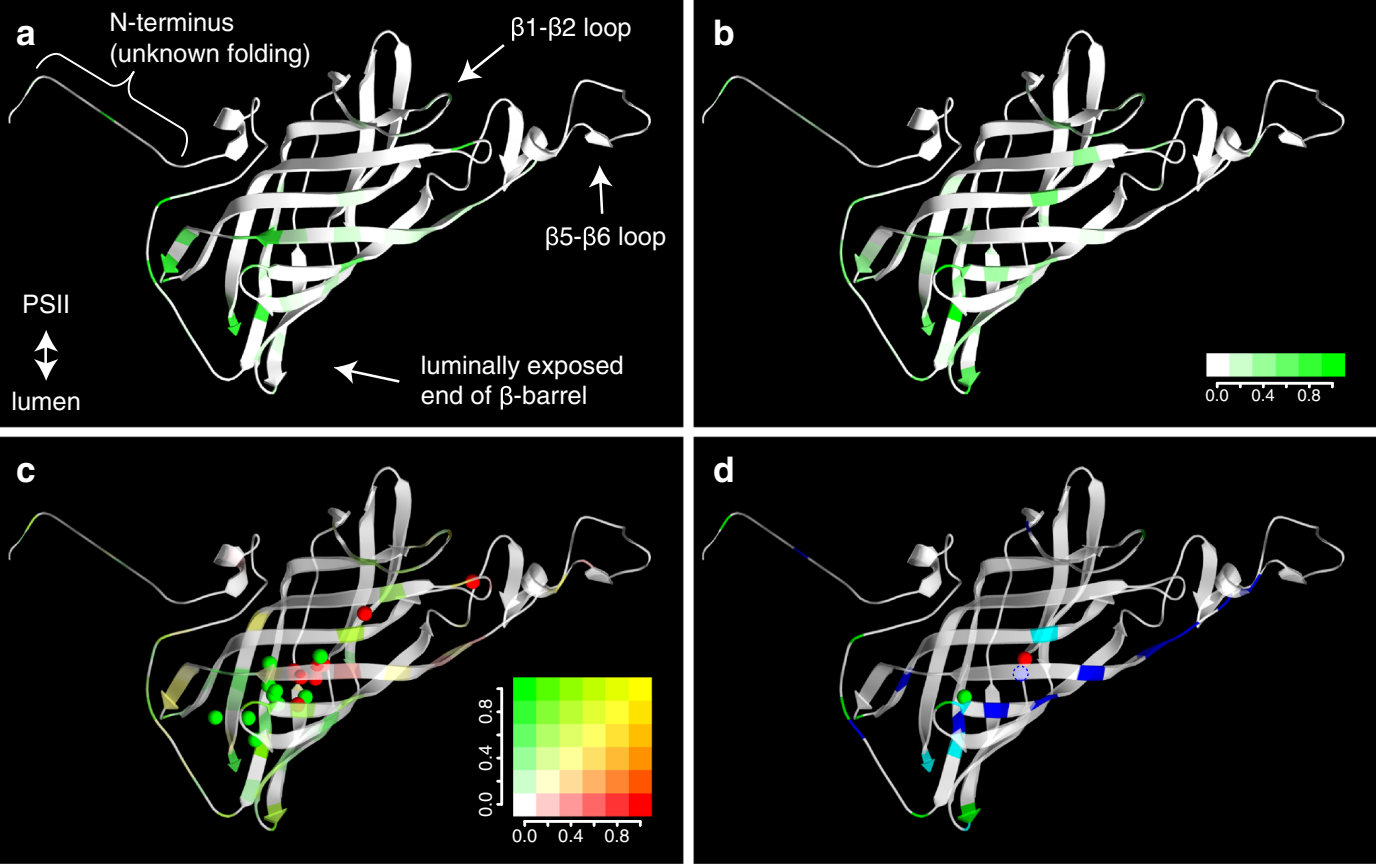
Mapping variable amino acid residues on the PsbO structure. **a** Differences between isoforms in Solanaceae species and (**b**) differences between isoforms averaged across all angiosperm families. The varying positions are green-coloured depending on frequency of differences among the analysed pairs of isoforms. **c** Merged differences between isoforms (in green) and between species (in red) with equally coloured spheres indicating spatial centres of these differences calculated separately for each angiosperm family, the frequency of particular differences on each position is indicated by colour gradient. **d** Merged averaged differences between isoforms (in green), between plant families (in blue) or both types (in cyan); only positions with a value of variability above a given threshold (0.24) are shown together with overall spatial centres of differences between isoforms, species (within families) and families (green, red and blue spheres, respectively). The homology model of the *Solanum tuberosum* PsbO2 based on the X-ray structure of cyanobacterial PsbO [PDB:3ARC] [5] was constructed using Swiss-Model program [38]; the first 13 N-terminal amino acids were not present in the template structure, so they were pasted in the model without attempts to show any folding and they were not included in calculation of the spatial centres

The isoforms diverged independently in every family, so we first mapped the isoform differences on the model in each family separately. Fig. 4a shows the model of PsbO coloured according to the frequency of differences between isoforms in species of the Solanaceae family. The differences are situated mostly on the luminal end of the β-barrel structure and some differences can be found also on the β1-β2 loop. Comparing this location with positions of differences between isoforms averaged across all angiosperm families, we can see that the general pattern is shared (Fig. 4b). Interestingly, the same pattern is exhibited also in the recently diverged isoforms of maize with only 6 different amino acids and in the moss *Physcomitrella patens* with four PsbO isoforms (Additional file 7).

Before drawing any conclusions, we had to prove that this spatial location is specific for differences between isoforms and does not reflect a high level of general variability in these regions. We compared the position of isoform differences with between-species differences in all families (Fig. 4c). We found that differences between species (red-coloured in the figure) are more dispersed over the PsbO structure. To allow statistical analysis, we calculated spatial centres of between-isoform differences and between-species differences (green and red spheres in Fig. 4c, respectively) for each family. The spatial centres of isoform differences are shifted towards the luminal end of the β-barrel (with one exception, the Salicaceae family, which has the centre of differences between isoforms shifted towards the β1-β2 loop due to high frequency of differences in this part of the structure). The shift of spatial centres of isoform differences compared with the centres of between-species differences is significant according to a randomization test (*p* = 0.002).

### PSII-exposed surface is conserved, while differences between families are mainly on the luminal side of the β5-β6 loop

**Fig. 5:**
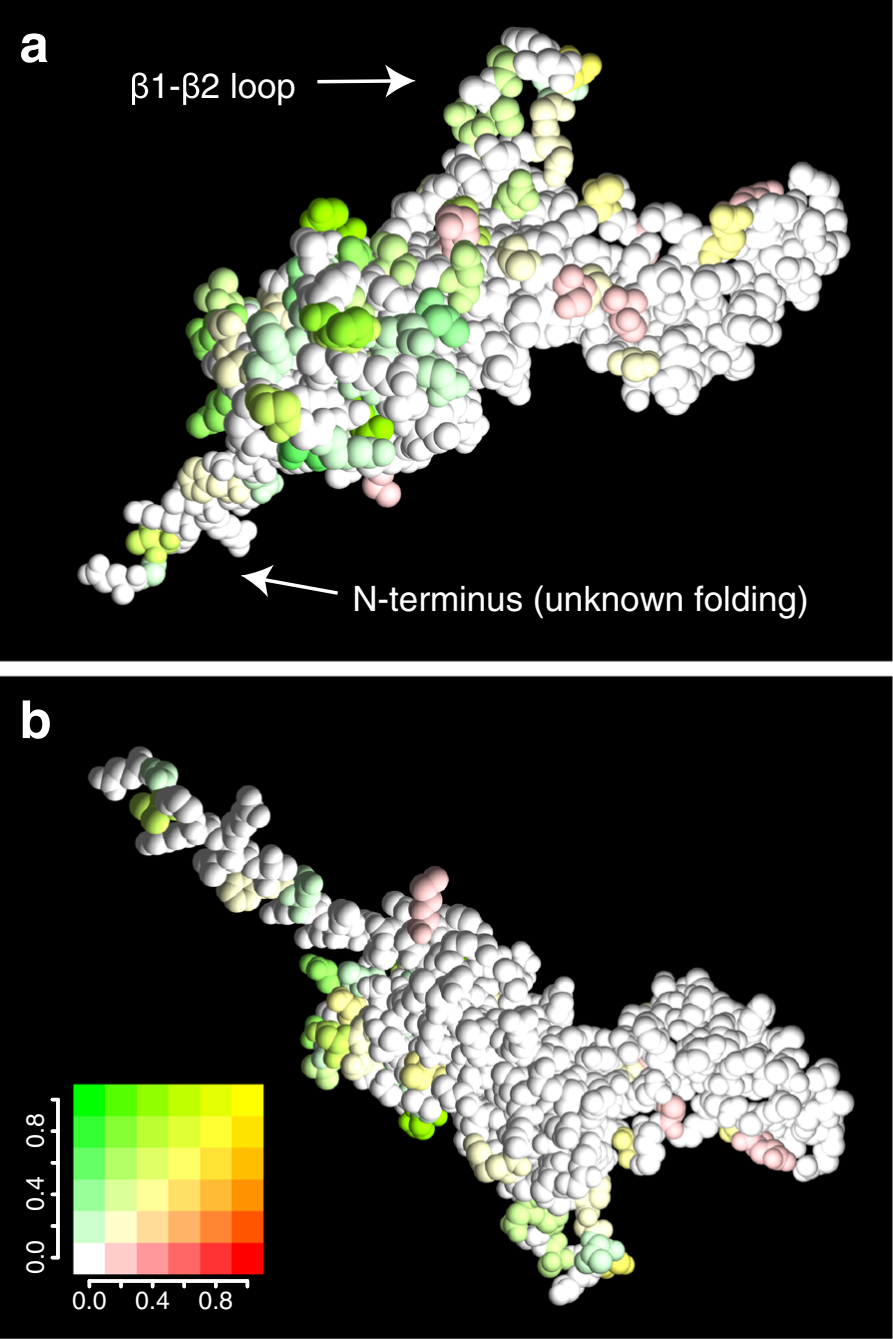
Mapping variable amino acid residues on the PsbO structure. **a** View from thylakoid lumen, (**b**) view from PSII. Differences between isoforms (in green) are merged with differences between species (in red); the frequency of particular differences on each position is indicated by colour gradient. The homology model of the *Solanum tuberosum* PsbO2 based on the X-ray structure of cyanobacterial PsbO [PDB:3ARC] [5] was constructed using Swiss-Model program [38]; the first 13 N-terminal amino acids were not present in the template structure, so they were pasted in the model without attempts to show any folding

Mapping of all variable positions on the model of PsbO structure also showed that the PsbO surface interacting with PSII core proteins is fully conserved in angiosperms with the exception of the β1-β2 loop (see Fig. 5). β1-β2 loop interacts with CP47 protein from the other monomer of PSII [5, 9].

Differences between families are the most frequent class of differences among PsbO sequences (Additional files 2 and 5). Fig. 4d depicts differences between families merged with the differences between isoforms and overall spatial centres of the three classes of differences (represented with green, red and blue spheres). The differences between families are more spread over the PsbO structure compared to the differences between isoforms, similarly to the differences between species within families. The highest frequency of differences between families is in the part of β5-β6 loop that is not interacting with PSII core proteins (the amino acid side chains are pointing towards thylakoid lumen) and the adjoining part of the β6 strand.

## Discussion

### Mechanism of duplication and subfunctionalisation of *psbO*

Several studies demonstrated that *A. thaliana* expresses two *psbO* paralogs [17–19, 21]. Here we show that *A. thaliana* is not an exception and that species from 9 out of 15 investigated angiosperm families also express two distinct *psbO* genes (Additional file 3). Unexpectedly, the phylogenetic analysis revealed that the *psbO* gene was not duplicated in the common ancestor of angiosperms, but the duplication occurred many times independently in individual families (Fig. 1).

There are various mechanisms by which gene duplication can occur. In terms of its extent, duplication can involve single genes, larger segments, chromosomes or entire genomes [41]. In *A. thaliana* and *Populus trichocarpa* we found that chromosomal segments containing *psbO* paralogs are collinear (i.e. contain homologous genes in a similar order; Duchoslav, Vosolsobě, and Fischer, unpublished results), which suggests that *psbO* was duplicated within the context of a larger-scale duplication.

The phylogenetic tree topology indicates that the duplication event occurred in ancestors of numerous families prior to extensive species radiation. The radiation that involved many extant plant lineages in Paleogene, was likely facilitated by the whole genome duplications (WGD) dated to the last global extinction period at the Cretaceous–Paleogene boundary about 66 million years ago [42, 43]. Based on this indirect evidence, we suggest that the *psbO* duplication was not gene specific, but rather that the paralogs were in many cases retained after WGD events that occurred independently in ancestors of many successful angiosperm families.

After WGD, most duplicated genes gradually accumulate deleterious mutations and vanish from the genome (within millions of years). More rarely the duplication leads to neo- or subfunctionalisation of the paralogs if these changes improve fitness [41]. Currently, one of the best models explaining stabilisation of duplicated genes is the EAC model of subfunctionalisation (escape from adaptive conflict) [44] based on the fact that a single protein can perform multiple catalytic or structural functions. In such case, the selective optimization of one function may lead to a decline in another function, creating an adaptive conflict that preserves the single copy gene/protein in an intermediate state. Casual gene duplication can provide a solution – escape from the adaptive conflict *via* functional specialisation of the resulting paralogs [41].

Multiple angiosperm species contain just two *psbO* paralogs with similar extent of diversification, so we assume that the presence of two different PsbO proteins gives an advantage to these species. Although many plants prosper with a single PsbO gene, in species with two isoforms, the loss of one isoform negatively affects growth and photosynthesis, e.g. in *A. thaliana* [18, 19, 23, 45] or potato [25]. It indicates that functions of current diversified PsbO isoforms are no more equivalent due to subfunctionalisation after the duplication.

### Structural aspects in PsbO diversification

Protein functions are connected with protein structure. Therefore, identification of common structural differences between isoforms in multiple species can indicate common functional adaptation. If various plants used duplicated *psbOs* to solve the same adaptive conflict, the structural and functional differentiation of PsbO isoforms would be similar or identical irrespective of independent duplication in individual families.

To evaluate the between-isoform differences, we first divided the overall variability of PsbO sequences on each position of the primary structure into three classes. The variability in current PsbO sequences reflects both differences present already in the ancestor species before *psbO* duplication (*between-families variability*) and differences obtained after the duplication, including specific diversification of isoforms (*between-isoforms variability*) and species-specific changes (*between-species variability*; Fig. 2; see quantification bellow the alignment in Additional file 2). The frequency of each variability class on specific positions was mapped on the homology model of PsbO (Fig. 4). The model corresponded to other published homology models of higher plants’ PsbO [9, 10]. The mapping showed that occupancy of the differences on the PsbO surface was unequal and the locations of the three classes of variability significantly differ.

There are practically no differences between isoforms on the PSII-binding surface of PsbO (Fig. 5). However, Murakami *et al.* [18] reported that PsbO2 of *A. thaliana* is less efficient in reconstitution of oxygen evolution *in vitro* compared to PsbO1. Our analysis showed that the PSII-binding surface is highly conserved in all angiosperms. It indicates that the differences in water oxidation observed by Murakami *et al.* [18] were not caused by direct modulation of water oxidation on Mn_4_CaO_5_ cluster, but rather by some indirect effect.

The biggest contrast in localisation of between-isoform differences and other types of differences (between-family and between-species) is in the part of β5-β6 loop that is not interacting with PSII core proteins (the amino acid side chains are pointing towards lumen) and the adjoining part of the β6 strand. In this part of PsbO, there is a very high frequency of between-family differences and a high frequency of between-species differences, whereas between-isoform differences are nearly absent. This suggests that this part of the PsbO surface might be involved in binding of some other protein, whose interaction surface can differ in individual species or families. As isoforms do not differ in this region, it seems that both isoforms need to retain this interaction identical. The presence of a hypothetical interactor is further supported by the fact that an unassigned density was detected in this part of PSII supercomplex structure by cryo-electron tomography [8].

The between-isoform differences were located mostly at the end of the β-barrel protruding into the lumen and on the β1-β2 loop. This pattern was similar in all analysed families and even in the moss *Physcomitrella* and the relatively recently duplicated *psbO* in maize (Fig. 4, Additional file 7). This indicates that the differences between isoforms probably enabled the same or similar functional adaptation of PsbOs in all analysed families. Since the *psbO* duplications were independent, the functional divergence of PsbO isoforms likely represents a parallel evolution, further supporting the impact of observed diversification of PsbO isoforms.

### Functional differences between PsbO isoforms

We found that the location of the differences between PsbO isoforms of *A. thaliana* fits the pattern found in other angiosperms. Nine out of 11 different amino acids are located at the luminal base of β-barrel and one is located on the β1-β2 loop (Additional file 7). Both PsbO isoforms of *A. thaliana* are able to stabilise the manganese-calcium cluster and enable water splitting [18, 19]. PsbO1 was demonstrated to provide more efficient water splitting [18], whereas PsbO2 was reported to have higher GTPase activity and was proposed to participate in D1 repair cycle [19, 21, 23].

The highest frequency of between-isoform differences is located just around the hypothetic GTP-binding site predicted by Lundin *et al.* [10], which is situated inside the luminal end of the β-barrel. Lundin *et al.* found hypothetic non-canonical GTP-binding domains in spinach [10] and *A. thaliana* PsbO sequence [21]. G1 domain, binding α-phosphate, was predicted in β1 sheet, G2-G3 domain, binding γ-phosphate, in β2 sheet and G4 domain, binding guanine ring, in β4-β5 loop (marked in the alignment in Additional file 2). Regions surrounding the G2-G3 domain, i.e. β1-β2 and β2-β3 loops, were predicted to be Switches I and II, respectively. These switches could have different conformations in GDP- and GTP-bound state.

The proposed G2-G3 domain is rather conserved in angiosperms, whereas G1 and G4 domains and Switches I and II have high frequency of differences between isoforms. The position with the most frequent differences between isoforms (T46 in spinach PsbO) is located in G1 domain, the position with the second most frequent differences between isoforms (E139 in spinach PsbO) is in G4 domain (see positions 47 and 140 in alignment in Additional file 2).

Since PsbO isoforms in *A. thaliana* were found to differ in GTPase activity [21] and the location of differences between them corresponds to the situation in other species, we propose that, in general, the differences between isoforms might modulate the GTPase activity of PsbO.

It is important to mention that the amino acids varying between isoforms have side chains mostly pointing outside of the β-barrel and not inside towards the predicted GTP-binding site. However, these amino acids might not only change the binding of GTP, but also modulate accessibility of the binding site for GTP or binding of some regulatory protein. Consistently with this assumption, we found that amino acid residues with longer side chains often predominated in one isoform, whereas residues with shorter chains in the other (mainly in the case of chains with interaction-competent carboxylic groups). Though the substitution between glutamic and aspartic acid is often regarded to be nearly synonymous, these residues alternate within isoform pairs very regularly on some positions. Their strong conservation in orthologous isoforms within families (and in some cases even across more families) indicates that they can affect the PsbO function. The simple increase in the length of amino acid side chain can allow the protein to keep the structure, isoelectric point and other important properties unchanged, whereas it can strongly facilitate (affect) the interactions with other proteins, e.g. PsbP, another extrinsic protein of PSII. Interestingly, PsbP was reported to have a crystal structure similar to the GTPase Ran regulatory protein [21, 46]. The importance of certain carboxylic amino acid residue in PsbO can be demonstrated on spontaneously tuberising potato mutant, where the absence of one PsbO isoform could not be complemented with another allele with a point mutation substituting glutamic acid for aspartic acid (Fischer and Duchoslav, unpublished results; [25]).

Based on the situation in *A. thaliana* and suggested isoform evolution by the “escape from adaptive conflict” scenario, we can hypothesise that the amino acid substitutions adapt the isoform to its specific function, which is obviously connected with losing in the other of the two functions. Murakami et al. [18] tested efficiency of *in vitro* oxygen evolution with native PsbO isoforms and their chimers that combined fragments (or selected amino acids) from both *A. thaliana* isoforms. They found that the weaker performance of PsbO2 was connected mainly with the C-terminal third of the protein, namely with two substitutions, V186S (in β5-β6 loop) and L246I (with side chain pointing towards the hypothetical GTP-binding site). On the contrary, substitution V204I (also with side chain pointing towards the hypothetical GTP-binding site) improved the activity and obviously compensated for the decrease in oxygen evolution caused by substitutions on the positions 186 and 246. Unfortunately, the GTPase activity of PsbO was proposed later, so the activity was not determined in these chimeric proteins to test the trade-off hypothesis. The importance of the C-terminal part (often differing between isoforms) was also demonstrated in spinach PsbO. The last three amino acid residues, including leucin at position 246 (245 in spinach), were shown to be critical for binding to PSII and for restoration of oxygen evolving activity [47].

Besides the modulation of the GTPase activity, there are some other presumptive interactions of PsbO that can be affected by the observed differences between isoforms. An obvious possibility is modulation of interaction with PsbP (mentioned above) or PsbQ, the other extrinsic proteins of PSII. The extrinsic proteins of PSII were suggested to be involved in the interaction between the thylakoid membranes across the lumen [12, 48]. Thus, the changes on the luminal end of the β-barrel of PsbO might modulate this interaction regardless whether the interaction is direct (PsbO-PsbO) or via other extrinsic proteins. Another possibility is that PsbO is interacting with some protein bound to the same membrane through the luminal end of the β-barrel, which is protruding not only to the lumen but also slightly over the outer rim of PSII towards the strongly bound (S) LHCII trimer [49]. This speculation is supported by the evidence that removal of PsbO has an effect on the position of LHCII in PSII-LHCII supercomplex [50] and that there is an unassigned density on the luminal side of the LHCII trimers in cryo-electron tomography structure of the supercomplex [8]. The N-terminus of PsbO might also be involved in such interaction, because angiosperm PsbOs are longer by usually 13 amino acids compared to the cyanobacterial protein analysed by Umena *et al.* [5]. Numerous biochemical studies were focused on site-directed mutagenesis of PsbO (reviewed in ref. [51] and [11]). However, these studies cannot be used for interpretation of our results, since the mutated residues were mostly the highly conserved ones.

### Speculative model on the role of PsbO isoforms

Our findings together with literature data led us to formulate a speculative model explaining the functional specialisation of PsbO isoforms. Hong *et al.* [52] showed that the oxidation state of the Mn_4_CaO_5_ cluster influences the structure of PsbO. We suggest that when the D1 protein and Mn_4_CaO_5_ cluster get damaged, PsbO shifts in the GTPase cycle. This induces a change in conformation of the β1-β2 loop. As the β1-β2 loop is interacting with the other monomer of PSII in the PSII dimer [9], such change might induce monomerisation of PSII [10] and its release from the semi-stabile organization in grana that allows diffusion to stroma lamellae. In stroma lamellae, PsbO released from PSII changes the GTPase state and D1 is degraded [53]. After reassembly of PSII, PsbO binds back.

We propose that the PsbO1 isoform is adapted for low light conditions. It has lower GTPase activity [21] (or lower affinity to GTP) and thus it does not induce as rapid monomerisation of PSII and exchange of damaged D1 subunit as the PsbO2 isoform. It was shown that under low irradiance, a part of PSII-LHCII supercomplexes form ordered crystalline arrays [54] and the grana are tightly stacked [55]. Thus, PsbO1 might prevent premature disturbance of the effectively working crystalline arrays of PSII-LHCII supercomplexes and support tight stacking of thylakoid membranes. We also suggest that PsbO2 is, on the other hand, optimized for high light conditions, when efficient light harvesting is not important due to the excess of energy, but the repair of damaged D1 subunits must be very fast to prevent further damage of photosynthetic complexes. Correspondingly, Herbstová *et al.* [56] demonstrated that high light treatment leads to an increase of protein mobility in grana thylakoids. Moreover, the proportion of PSII in PSII-LHCII supercomplexes in *A. thaliana* is high in *psbo2* mutant and low in *psbo1* mutant compared to wild-type plants [21]. Thus, the fast GTPase activity (or high affinity to GTP) of PsbO2 might facilitate rapid disorganisation of PSII-LHCII supercomplexes and crystalline arrays allowing rapid diffusion of damaged PSII out of the grana and their redeployment after D1 replacement. Modulation of the ratio of PsbO1 and PsbO2 bound to PSII can help to adjust and optimise the photosynthetic performance in response to light conditions. An increase in the amount of PsbO2 in *A. thaliana* after long-term cold stress [22], which is in many features similar to high light stress [57], supports our speculation. It suggests that the PsbO1/2 availability is regulated transcriptionally in the long term. The momentary binding of isoforms might be regulated, for example, by different dynamics of the GTPase cycle.

In the species with single PsbO isoform, the function of the PsbO is likely in the mid-way between the two specialised isoforms present in other species and supports both of the specialised functions, at least at the basic level. Depending on the growth strategy and habitat, the single isoform could be principally closer to *A. thaliana* PsbO1 (shade plants), to PsbO2 (sunny plants) or intermediate. For example, PsbO protein sequences of Poaceae species are closer to PsbO2 than to PsbO1 of *A. thaliana*, especially regarding the positions with alternating glutamic and aspartic acid residues (Additional files 2 and 6), which is consistent with the typical sunny habitat of these species. However, the species with single isoform that cluster to *A. thaliana* PsbO1 cannot be considered as shade plants. Consequently, the isoform sequence cannot be simply related to the actual habitat certain species, but it is obviously also affected by other factors such as evolutionary history or life strategy. Nevertheless, we can conclude from these considerations that *A. thaliana* PsbO1 and its functional analogs cannot be simply assumed as the main isoforms, because the importance of certain isoform depends on the habitat or even on actual growth conditions.

## Conclusions

Our study showed that the pairs of PsbO isoforms evolved in numerous angiosperm lineages independently. Yet, the pairs of PsbO isoforms differ at similar regions in the protein structure, mostly on the luminally exposed end of the β-barrel structure near the predicted GTP-binding site. Mapping of conserved and variable positions on PsbO surface also indicated new potential interaction regions. Observed analogy in divergence between isoforms in various species indicates that structural diversification and subfunctionalisation of PsbO isoforms represents an example of parallel evolution. Features evolved in parallel likely bring a significant advantage, so we assume that diversification of PsbO isoforms improve photosynthetic performance under variable conditions. However, the predicted subfunctionalisation related to diverse GTPase activity will require further experimental confirmation.

### Availability of supporting data

The phylogenetic data used in this study have been deposited in TreeBASE database (http://purl.org/phylo/treebase/phylows/study/TB2:S17601).

## Additional files

Additional file 1:
**List of analyzed** ***psbO*** **genes.** The dataset includes gene names used in the study, accession numbers, source databases and cDNA sequences. Additional file 2:
**Alignment of the protein sequences of mature PsbOs from analyzed angiosperm species.** The calculated values of the differences between isoforms, the differences between species and the differences between families are shown below the alignment. The sequences that were not included in the calculation are marked with an asterisk. Hypothetic GTP-binding domains (G motifs) predicted by Lundin *et al.* [10], conserved regions identified in wide range of photosynthetic organisms by De Las Rivas and Barber [9] and β-sheets forming the β-barrel structure are indicated below the alignment. Horizontal lines separate sequences from species belonging to the same angiosperm family. Additional file 3:
**Number and divergence of expressed PsbO isoforms in the analysed land plant species.** Number of distinct *psbO* cDNA sequences found in EST databases and maximal number of different amino acid residues in mature proteins derived from these sequences. Additional file 4:
**A phylogenetic tree from coding sequences of** ***psbO*** **genes from 49 land plant species.** The tree was constructed by the maximum likelihood method, numbers at branches denote bootstrap percentages. Additional file 5:
**Venn diagrams of amino acid positions clustered according to the predominant class of variability.** Threshold values of each type of variability to include an amino acid position in the diagrams are indicated. Additional file 6:
**Numbers of glutamic and aspartic acid residues in PsbO protein sequences.** Each point represents one PsbO; isoforms from one species are connected with a line, filled circles represent PsbOs from species with only one isoform. Points with the same coordinates are slightly shifted in order to make them visible. One representative species with two isoforms is shown from each family. Plotted species: *Arabidopsis thaliana* (Ath), *Artemisia annua* (Aan), *Citrus sinensis* (Csi), *Eucalyptus grandis* (Egr), *Fragaria vesca* (Fve), *Gossypium raimondii* (Gra), *Hordeum vulgare* (Hvu), *Linum usitatissimum* (Lus), *Lotus japonicus* (Lja), *Malus domestica* (Mdo), *Manihot esculenta* (Mes), *Mimulus guttatus* (Mgu), *Oryza sativa* (Osa), *Populus trichocarpa* (Ptr), *Solanum tuberosum* (Stu), *Spinacia oleracea* (spinach, Sol), *Theobroma cacao* (Tca), *Triticum aestivum* (Tae), *Vitis vinifera* (Vvi), *Zea mays* (Zma), *Zingiber officinale* (Zof). Additional file 7:
**Mapping differences between isoforms on PsbO structure in selected species.** Differences between isoforms of (A) *A. thaliana*, (B) *Zea mays* and (C) *Physcomitrella patens* are shown in green. The homologous model of the *Solanum tuberosum* PsbO2 based on the X-ray structure of cyanobacterial PsbO [PDB:3ARC] [5] was constructed using Swiss-Model program [38]; the first 13 N-terminal amino acids were not present in the template structure, so they were pasted in the model without attempts to show any folding.
