# Supplementary material for: Parallel subfunctionalisation of PsbO protein isoforms in angiosperms revealed by phylogenetic analysis and mapping of sequence variability onto protein structure
Source: BMC Plant Biol. 2015 Jun 9;15:133. doi: 10.1186/s12870-015-0523-4 (PMC4459440; doi:10.1186/s12870-015-0523-4)
Supplement: Additional file 3: — Number and divergence of expressed PsbO isoforms in the analysed land plant species. Number of distinct psbO cDNA sequences found in EST databases and maximal number of different amino acid residues in mature proteins derived from these sequences. [file 12870_2015_523_MOESM3_ESM.pdf]

Additional file 3: Number and divergence of expressed PsbO isoforms in the analyzed land plant species. Number of distinct *psbO* cDNA sequences found in EST databases and maximal number of different amino acid residues in mature proteins derived from these sequences.

	Family	Species	Number of PsbO isoforms	Maximal number of different amino acid residues between any two isoforms[a]
Bryophyta	Funariaceae	*Physcomitrella patens*	4	24
Lycopodiophyta	Selaginellaceae	*Selaginella moellendorffii*	1[b]	
Cycadophyta	Cycadaceae	*Cycas rumphii*	1	
Coniferophyta	Cupressaceae	*Cryptomeria japonica*	1	
		Sequoia sempervirens	1	
	Pinaceae	*Picea abies*	1	
		Picea sitchensis	1	
		Pinus sylvestris	1	
		Pinus taeda	1	
	Taxaceae	*Taxus baccata*	1	
monocots	Poaceae	*Hordeum vulgare*	1	
		Oryza sativa	1	
		Triticum aestivum	1	
		Zea mays	2	6
	Zingiberaceae	*Curcuma longa*	2	13
		Zingiber officinale	2	12
dicots	Asteraceae	*Artemisia annua*	2	13
	Brassicaceae	*Arabidopsis thaliana*	2	11
		Brassica napus	5	7
		Brassica oleracea	3	9
		Brassica rapa	4	7
		Thellungiella halophila	3	10 (41)[c]
	Euphorbiaceae	*Manihot esculenta*	2	14
	Fabaceae	*Glycine max*	4	13
		Lotus japonicus	2	15
		Medicago truncatula	2	12
		Phaseolus vulgaris	2	7
	Linaceae	*Linum usitatissimum*	1[d]	
	Malvaceae	*Gossypium raimondii*	1	
		Theobroma cacao	1	
	Myrtaceae	*Eucalyptus grandis*	1	
	Phrymaceae	*Mimulus guttatus*	1	
	Rosaceae	*Fragaria vesca*	1	
		Malus domestica	2	11
	Rutaceae	*Citrus reticulata*	1	
		Citrus sinensis	1	
		Citrus trifoliata	1	
	Salicaceae	*Populus x canadensis*	2	23
		Populus deltoides	2	23
		Populus trichocarpa x Populus deltoides	2	22
		Populus trichocarpa	2	20
	Solanaceae	*Capsicum annuum*	2	18
		Nicotiana benthamiana	2	12
		Nicotiana tabacum	4	14
		Solanum habrochaites	2	15
		Solanum lycopersicum	2	13
		Solanum melongena	2	21
		Solanum tuberosum	2	12
	Vitaceae	*Vitis vinifera*	2	17

[a] in mature form (without a transite peptide)
[b] Two psbO sequences of *S. moellendorffii* can be found in EST databases. One of them (PlantGDB: PUT-165a-Selaginella_moellendorffii-12671) has far different mutation rate and seems to be a slightly expressed pseudogene, thus was omitted from analyses.
[c] Two isoforms of *T. halophila* that are most similar to isoforms of *A. thaliana* have only 10 different amino acid residues, another, the most diverged isoform, is orthologous to a truncated pseudogene of *A. thaliana*.
[d] *L. usitatissimum* expresses two psbO genes, but the corresponding mature proteins have the same amino acid sequence.
