# Supplementary material for: Parallel subfunctionalisation of PsbO protein isoforms in angiosperms revealed by phylogenetic analysis and mapping of sequence variability onto protein structure
Source: BMC Plant Biol. 2015 Jun 9;15:133. doi: 10.1186/s12870-015-0523-4 (PMC4459440; doi:10.1186/s12870-015-0523-4)
Supplement: Additional file 4: — A phylogenetic tree from coding sequences of psbO genes from 49 land plant species. The tree was constructed by the maximum likelihood method, numbers at branches denote bootstrap percentages. [file 12870_2015_523_MOESM4_ESM.pdf]

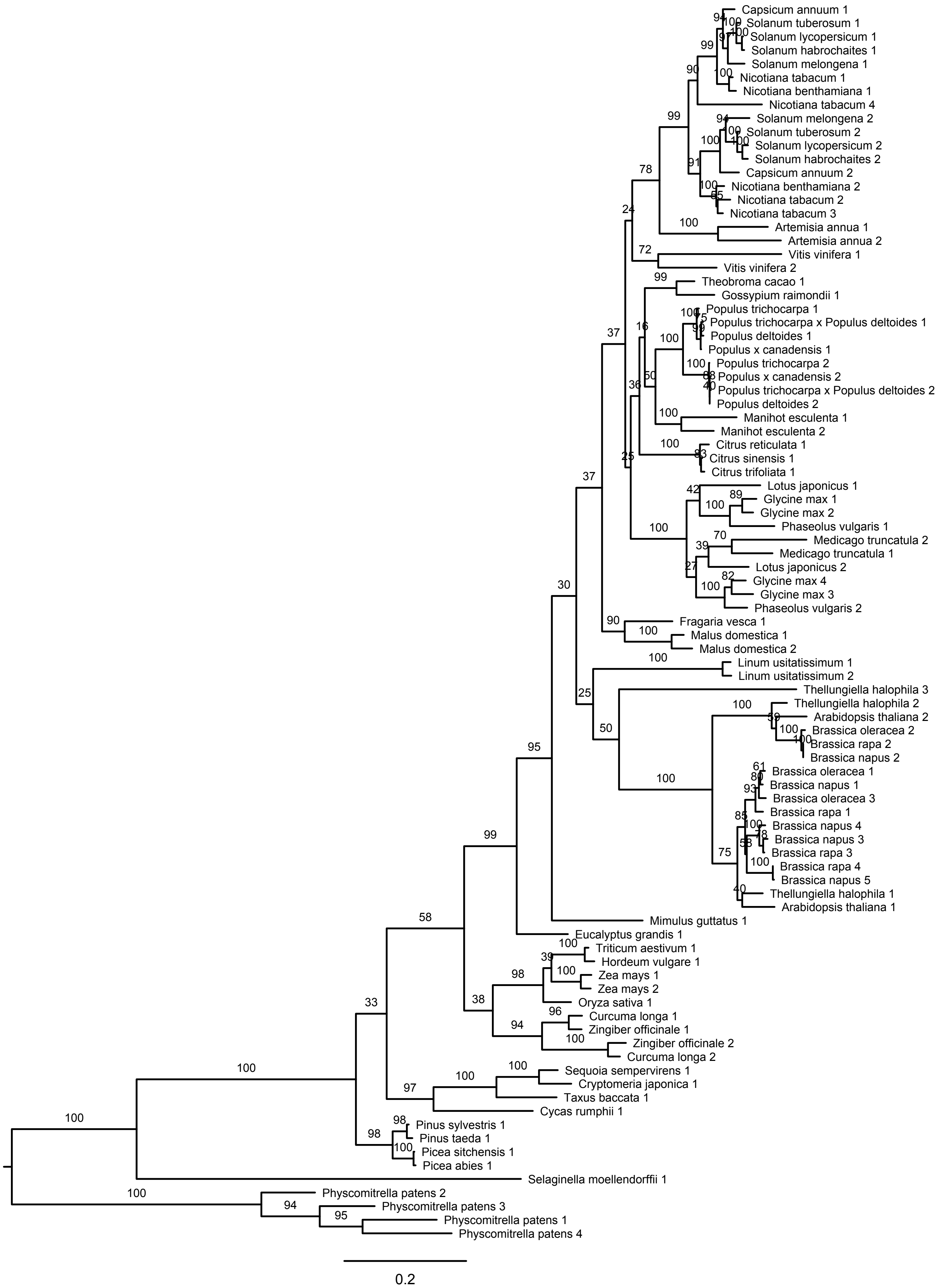

Additional file 4: A phylogenetic tree from coding sequences of *psbO* genes from 49 land plant species. The tree was constructed by the maximum likelihood method, numbers at branches denote bootstrap percentages.
