# Supplementary material for: Parallel subfunctionalisation of PsbO protein isoforms in angiosperms revealed by phylogenetic analysis and mapping of sequence variability onto protein structure
Source: BMC Plant Biol. 2015 Jun 9;15:133. doi: 10.1186/s12870-015-0523-4 (PMC4459440; doi:10.1186/s12870-015-0523-4)
Supplement: Additional file 5: — Venn diagrams of amino acid positions clustered according to the predominant class of variability. Threshold values of each type of variability to include an amino acid position in the diagrams are indicated. [file 12870_2015_523_MOESM5_ESM.pdf]

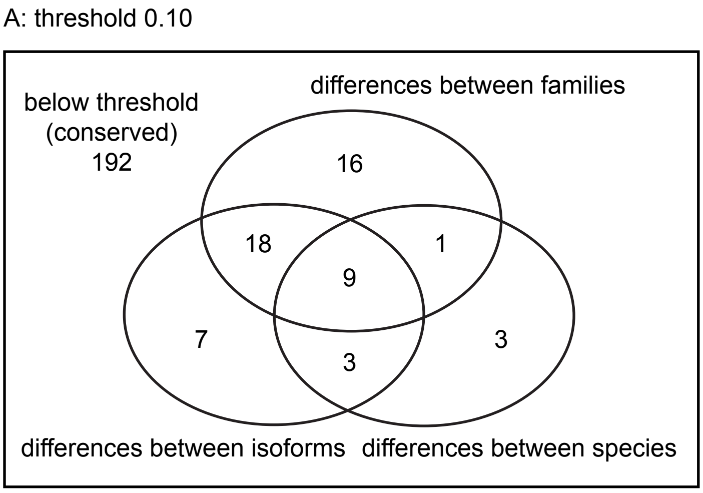

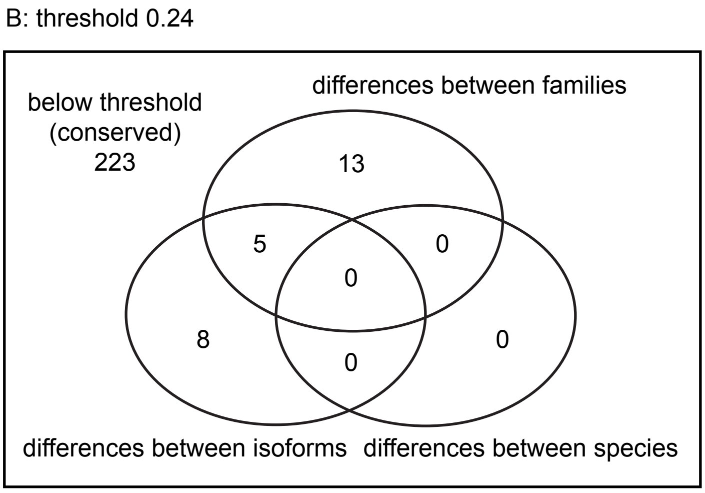

Additional file 5: Venn diagrams of amino acid positions clustered according to the predominant class of variability. Threshold values of each type of variability to include an amino acid position in the diagrams are indicated.
