# Supplementary material for: Parallel subfunctionalisation of PsbO protein isoforms in angiosperms revealed by phylogenetic analysis and mapping of sequence variability onto protein structure
Source: BMC Plant Biol. 2015 Jun 9;15:133. doi: 10.1186/s12870-015-0523-4 (PMC4459440; doi:10.1186/s12870-015-0523-4)
Supplement: Additional file 6: — Numbers of glutamic and aspartic acid residues in PsbO protein sequences. Each point represents one PsbO; isoforms from one species are connected with a line, filled circles represent PsbOs from species with only one isoform. Points with the same coordinates are slightly shifted in order to make them visible. One representative species with two isoforms is shown from each family. Plotted species: Arabidopsis thaliana (Ath), Artemisia annua (Aan), Citrus sinensis (Csi), Eucalyptus grandis (Egr), Fragaria vesca (Fve), Gossypium raimondii (Gra), Hordeum vulgare (Hvu), Linum usitatissimum (Lus), Lotus japonicus (Lja), Malus domestica (Mdo), Manihot esculenta (Mes), Mimulus guttatus (Mgu), Oryza sativa (Osa), Populus trichocarpa (Ptr), Solanum tuberosum (Stu), Spinacia oleracea (spinach, Sol), Theobroma cacao (Tca), Triticum aestivum (Tae), Vitis vinifera (Vvi), Zea mays (Zma), Zingiber officinale (Zof). [file 12870_2015_523_MOESM6_ESM.pdf]

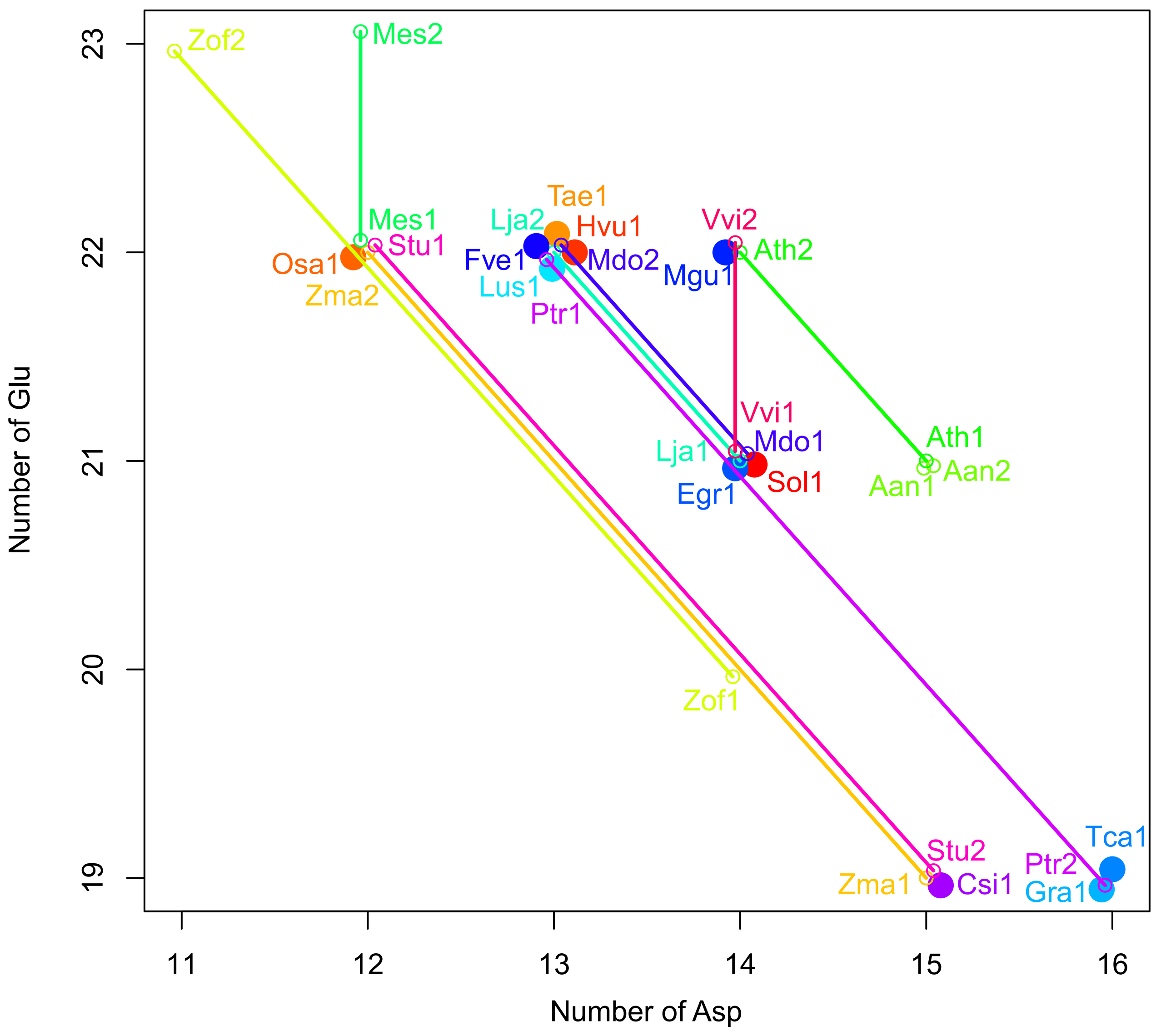

Additional file 6: Numbers of glutamic and aspartic acid residues in PsbO protein sequences. Each point represents one PsbO; isoforms from one species are connected with a line, filled circles represent PsbOs from species with only one isoform. Points with the same coordinates are slightly shifted in order to make them visible. One representative species with two isoforms is shown from each family. Plotted species: *Arabidopsis thaliana* (Ath), *Artemisia annua* (Aan), *Citrus sinensis* (Csi), *Eucalyptus grandis* (Egr), *Fragaria vesca* (Fve), *Gossypium raimondii* (Gra), *Hordeum vulgare* (Hvu), *Linum usitatissimum* (Lus), *Lotus japonicus* (Lja), *Malus domestica* (Mdo), *Manihot esculenta* (Mes), *Mimulus guttatus* (Mgu), *Oryza sativa* (Osa), *Populus trichocarpa* (Ptr), *Solanum tuberosum* (Stu), *Spinacia oleracea* (spinach, Sol), *Theobroma cacao* (Tca), *Triticum aestivum* (Tae), *Vitis vinifera* (Vvi), *Zea mays* (Zma), *Zingiber officinale* (Zof).
