# Supplementary material for: Parallel subfunctionalisation of PsbO protein isoforms in angiosperms revealed by phylogenetic analysis and mapping of sequence variability onto protein structure
Source: BMC Plant Biol. 2015 Jun 9;15:133. doi: 10.1186/s12870-015-0523-4 (PMC4459440; doi:10.1186/s12870-015-0523-4)
Supplement: Additional file 7: — Mapping differences between isoforms on PsbO structure in selected species. Differences between isoforms of (A) A. thaliana, (B) Zea mays and (C) Physcomitrella patens are shown in green. The homologous model of the Solanum tuberosum PsbO2 based on the X-ray structure of cyanobacterial PsbO [PDB:3ARC] [5] was constructed using Swiss-Model program [38]; the first 13 N-terminal amino acids were not present in the template structure, so they were pasted in the model without attempts to show any folding. [file 12870_2015_523_MOESM7_ESM.pdf]

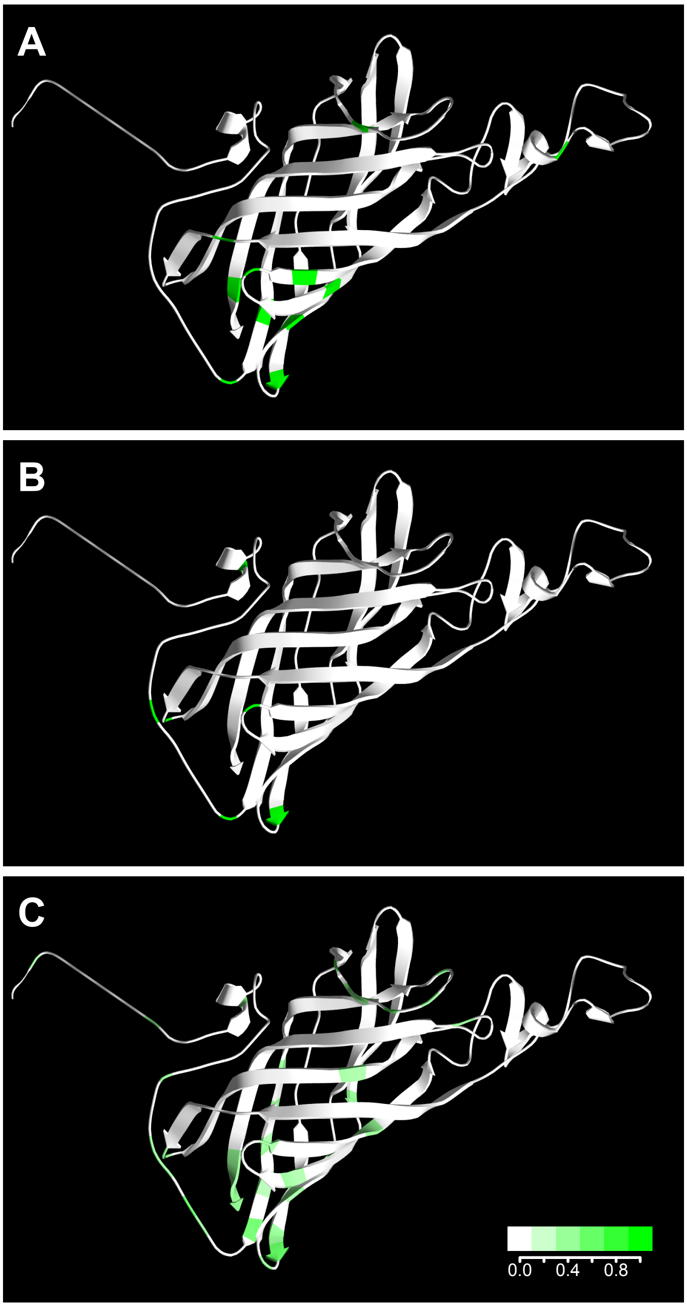


Additional file 7: Mapping differences between isoforms on PsbO structure in selected species. Differences between isoforms of (A) *A. thaliana*, (B) *Zea mays* and (C) *Physcomitrella patens* are shown in green. The homologous model of the Solanum tuberosum PsbO2 based on the X-ray structure of cyanobacterial PsbO [PDB:3ARC] [5] was constructed using Swiss-Model program [38]; the first 13 N-terminal amino acids were not present in the template structure, so they were pasted in the model without attempts to show any folding.
