# Supplementary material for: Parallel subfunctionalisation of PsbO protein isoforms in angiosperms revealed by phylogenetic analysis and mapping of sequence variability onto protein structure
Source: BMC Plant Biol. 2015 Jun 9;15:133. doi: 10.1186/s12870-015-0523-4 (PMC4459440; doi:10.1186/s12870-015-0523-4)
Supplement: Additional file 2: — Alignment of the protein sequences of mature PsbOs from analyzed angiosperm species. The calculated values of the differences between isoforms, the differences between species and the differences between families are shown below the alignment. The sequences that were not included in the calculation are marked with an asterisk. Hypothetic GTP-binding domains (G motifs) predicted by Lundin et al. [10], conserved regions identified in wide range of photosynthetic organisms by De Las Rivas and Barber [9] and β-sheets forming the β-barrel structure are indicated below the alignment. Horizontal lines separate sequences from species belonging to the same angiosperm family. [file 12870_2015_523_MOESM2_ESM.pdf]

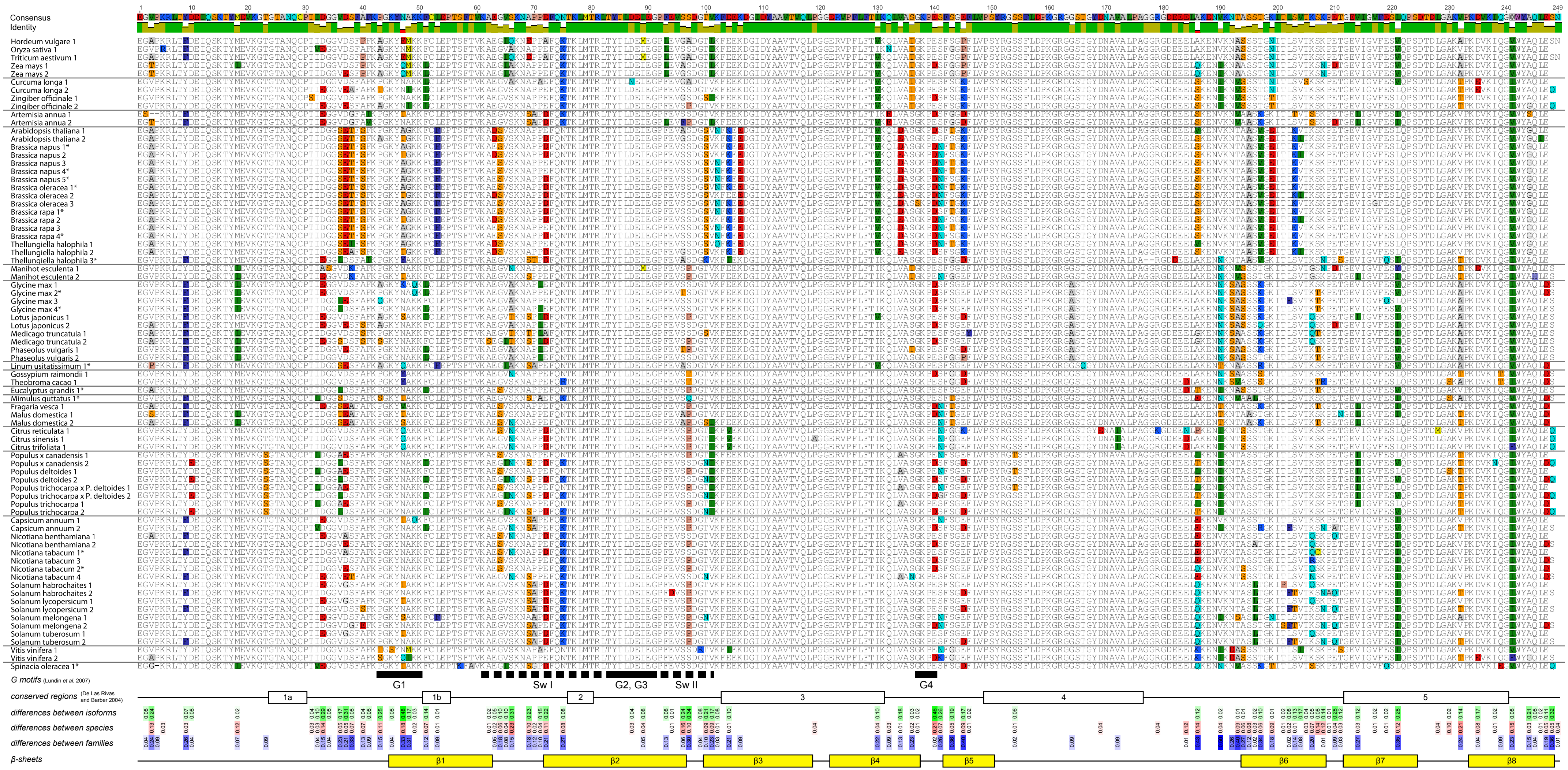

Additional file 2: Alignment of the protein sequences of mature PsbOs from analyzed angiosperm species. The calculated values of the differences between isoforms, the differences between species and the differences between families are shown below the alignment. The sequences that were not included in the calculation are marked with an asterisk. Hypothetic GTP-binding domains (G motifs) predicted by Lundin *et al.* [10], conserved regions identified in wide range of photosynthetic organisms by De Las Rivas and Barber [9] and β-sheets forming the β-barrel structure are indicated below the alignment. Horizontal lines separate sequences from species belonging to the same angiosperm family.
